# Establishing a robust multidisciplinary team process in complex abdominal wall reconstruction within a colorectal surgical unit

**DOI:** 10.1007/s10151-019-01965-4

**Published:** 2019-04-15

**Authors:** L. J. Muirhead, A. V. Shaw, C. Kontovounisios, O. J. Warren

**Affiliations:** 1 Department of Colorectal Surgery, Chelsea and Westminster Hospital, London, UK; 2 Department of Plastic Surgery, Chelsea and Westminster Hospital, London, UK; 3 Department of Surgery and Cancer, Imperial College, London, UK; 4 Department of Colorectal Surgery, Royal Marsden Hospital, London, UK

## Introduction

There is an increasing disease burden of complex abdominal wall herniation [1]. This is likely secondary to improved survival following intra-abdominal catastrophe, an ageing population and failure of primary hernia repair (both open and minimal access techniques). Colorectal surgeons continue to contribute to the problem; incisional hernia rates following midline laparotomy remain high at 22.4% at 3 years [2], and controversy remains regarding the best method of primary closure to prevent future herniation [3]. Even when strong evidence exists to support one type of primary closure over another, uptake of new techniques remains poor. Due to the nature of the pathologies and the procedures that often create the initial problem, these patients frequently find themselves in the colorectal surgical clinic.

Traditionally patients with complex abdominal wall defects have been managed by single-handed enthusiasts developing expertise, often in isolation, over a prolonged time period. Similar to cancer surgery a few decades ago, transparency with regard to activity levels, outcomes and resource utilisation has often been lacking. This despite major abdominal wall reconstruction (AWR) surgery necessitating complex decision making, frequently involving different surgical specialities, and being resource intensive with prolonged theatre times and hospital stays, and the use of expensive implants [4]. There is also increasing evidence that recording of surgical approach, mesh implant type and position, and patient outcomes within formal registries improve care [4].

Multidisciplinary team (MDT) management is increasingly the standard of care in many colorectal pathologies, both malignant and benign (e.g., inflammatory bowel disease). MDT management is not yet routine practise in complex AWR. We describe below our initial experience in establishing an MDT in complex AWR and propose a structure and some process and outcome measures to support any colorectal unit keen to do so.

## Results

Eighteen meetings were held over the first 36-month period (1st January 2016–1st January 2019). All consultants from the General Surgery and Plastic and Reconstructive Surgery Departments are welcome to attend and to refer patients to the MDT. Meetings are only considered quorate if at least one consultant from both of those departments is in attendance. One senior trainee from either speciality is always present and leads the meeting. We have achieved senior radiology input on only one occasion and having a regular radiology contribution remains a future goal, affected partly by funding. We have trialled patient involvement; two patients have been invited and attended two different MDT meetings with a family member or carer, to witness and participate in the discussion regarding their case. We remain keen to further understand how best to involve patients.

**Fig. 1:**
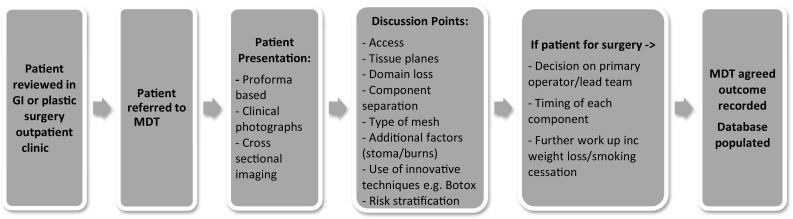
Process of AWR MDT

**Fig. 2:**
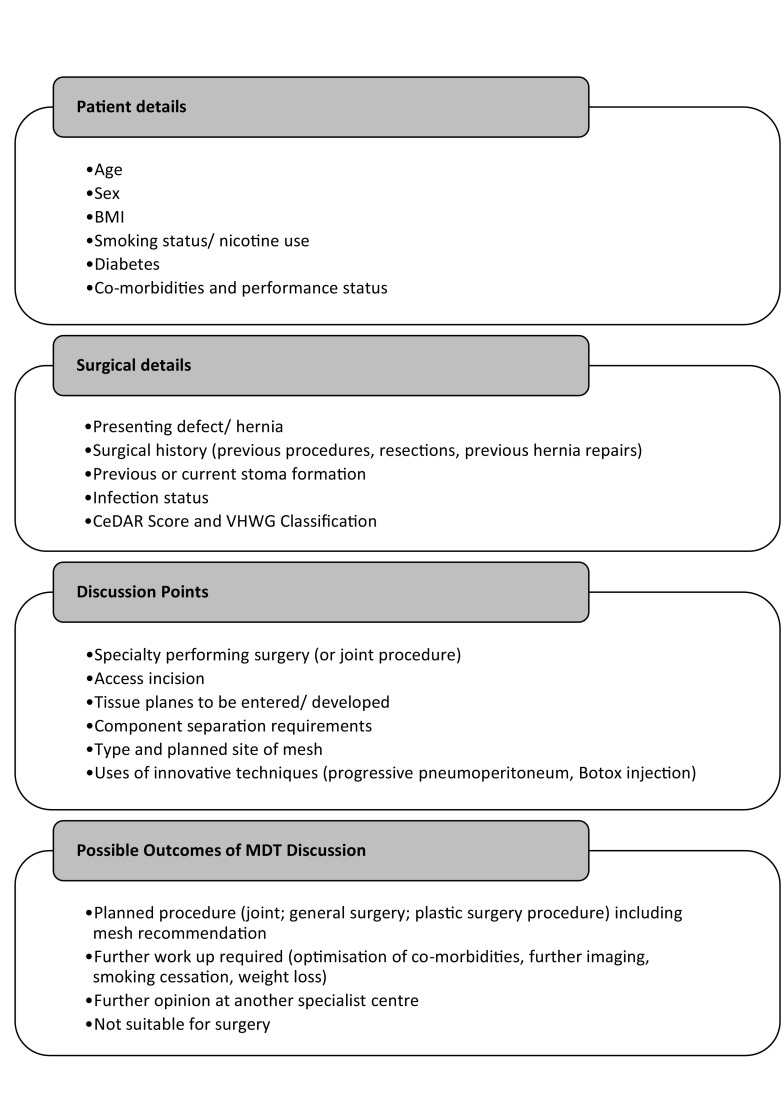
Current proforma for patient discussion

Figure 1 demonstrates the process. Prior to discussion in the MDT all patients must be seen by a consultant, and have three-dimensional (3D)imaging performed, most typically portal venous phase computed tomography (CT) scanning of the abdomen and pelvis. Where consent is given, high resolution medical photography is also employed pre-MDT. This gives high-quality reproducible images, allowing for better visualisation of the problem by those clinicians who have not met the patient. Patients are presented based on a proforma which has been developed over the last 3 years through an iterative process (see Fig. 2). Data are collected in real time on the discussions and is documented in the patients’ electronic medical records.

Seventy patients have been discussed (M:F 27:43). Mean age was 52 years (range 14–85 years). Mean body mass index (BMI) was 33.8 kg/m^2^ with 40% of patients having a BMI over 30 kg/m^2^. Twenty-two percent of patients were active smokers. Twelve percent had a diagnosis of diabetes (type 1 or 2).

Table 1 summarises the case mix discussed thus far in the meetings. The majority of cases are abdominal wall hernias secondary to previous surgery, laparostomy formation or extensive debridement. Additional complicating factors include multiple hernias, skin and soft tissue loss, previous or current infection, concurrent stoma, significant loss of domain, multiple previous mesh placements, and significant medical comorbidities.

**Table 1:**
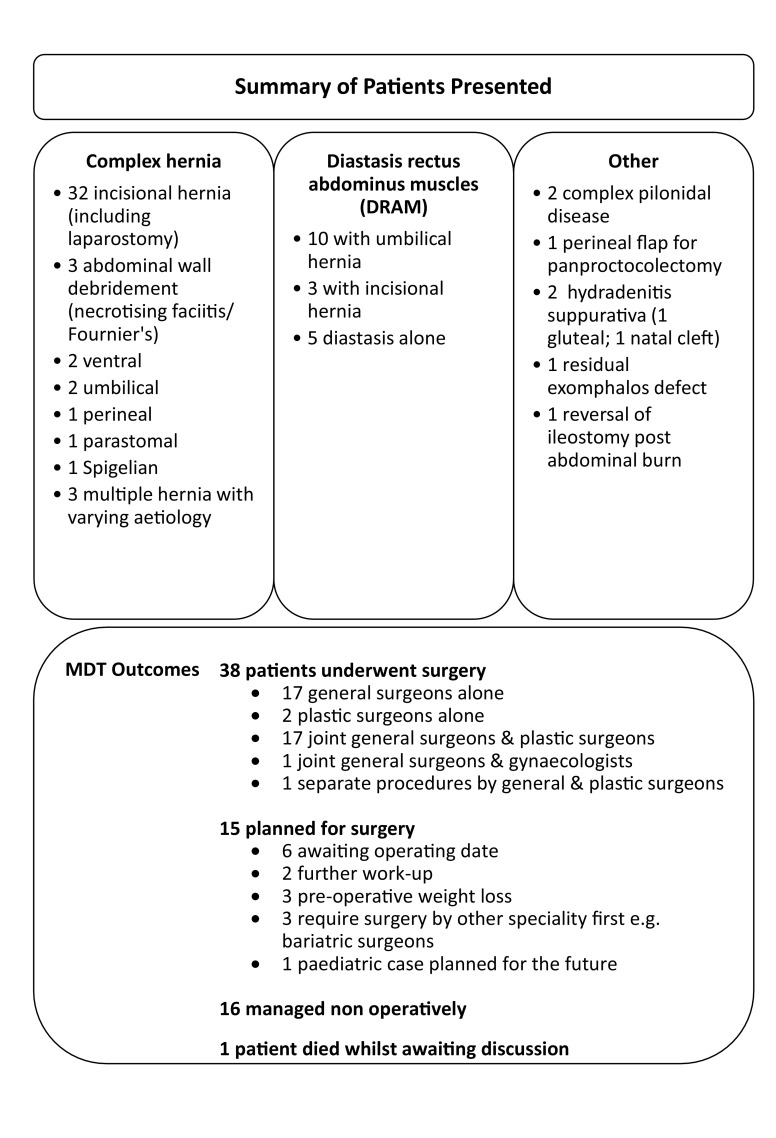
Patients presented 1st January 2016–1st January 2019

Following discussion, 38 patients underwent surgery; 17 procedures were performed by general surgeons alone, 17 were joint cases between plastic and general surgeons, and 2 were performed by plastic surgeons alone. One patient had 2 separate procedures performed by general and plastic surgeons independently and 1 patient had a joint procedure performed by general and gynaecological surgeons. In 34 of the 36 cases where a general surgeon was involved, this was a colorectal sub-specialist.

Fifteen patients are currently planned for surgery; 7 are awaiting an operating date, 2 require further work up, 3 require preoperative weight loss and 3 are awaiting surgery by another specialty (e.g., bariatric surgeons) first.

Sixteen patients have been managed non-operatively, usually as they were considered too high risk or no satisfactory operative interventions could be found. Two of these patients have been referred for consideration of an abdominal wall transplant. One patient died of consequences of hernia incarceration whilst waiting for discussion.

During this time period three new innovations have been introduced to the trust through the MDT process.

1. New surgical techniques—during the first 24 months of the MDT, we were unable to offer posterior component separation (PCS) with transversus abdominus release (TAR) as a treatment option for large complex ventral hernia. However, following appropriate education, cadaveric training and mentoring from colleagues outside the trust, this procedure has been offered and performed now on five patients by the senior author (OJW) with no major postoperative complications (Clavien-Dindo ≥ 2).
2. ‘Chemical component separation’, i.e., botulinum toxin injection into the lateral abdominal wall musculature prior to the repair of complex ventral hernia has been given to two patients. The first underwent injection in August 2016 followed by TAR in early October and the second underwent injection in January 2019 and is awaiting surgery shortly.
3. Creation of a regional network approach—two joint MDT meetings with colleagues at another London hospital who also have an AWR unit have occurred with a third one planned in March 2019.

## Conclusions

Most colorectal surgeons will not infrequently encounter patients with complex abdominal wall pathology, including multiple hernias, stomas, previous or current infection, loss of domain, and possibly pre-existing mesh. Managing these patients in isolation is increasingly difficult and exposes both patient and clinician to risk, including the possibility of making the situation worse.

We advocate the establishment of an MDT, regular meetings and the process that wraps around these. An MDT process brings benefits for both patient and clinician. It provides an opportunity for objective risk stratification and guidance regarding the need for prehabilitation interventions such as smoking cessation, weight loss and diabetic control. It allows the pooling of expertise and creates educational opportunities for both established surgeons and trainees. It promotes clinical governance of innovation and the introduction of new techniques and allows for prospective data collection on both recommended surgical approach and patient outcomes. This is key to understanding our own practice and ensuring consistency and quality of care.

Three years after we established an MDT in our organisation, we are still learning and see this as an iterative process. Prospective outcome data on every patient has started, as has the creation of networks of practise based on collaborative working across different providers and regions. Future possibilities for MDT outcomes include mesh recommendation on all patients, with deviation from this recommendation requiring surgeon explanation postoperatively, and the precise documentation of preoperative risk using scoring tools such as Americas Hernia Society Quality Collaborative (AHSQC) and Carolinas Equation for Determining Associated Risks (CeDAR) on all patients.
